# Pharmacogenetics of HIV therapy: State of the art in Latin American countries

**DOI:** 10.1590/1678-4685-GMB-2022-0120

**Published:** 2022-09-30

**Authors:** Camila de Almeida Velozo, Flávia Rachel Moreira Lamarão, Lucia Elena Alvarado-Arnez, Cynthia Chester Cardoso

**Affiliations:** 1 Universidade Federal do Rio de Janeiro, Instituto de Biologia, Laboratório de Virologia Molecular, Rio de Janeiro, RJ, Brazil.; 2 GlaxoSmithKline, Rio de Janeiro, RJ, Brazil.; 3 Universidad Franz Tamayo, Coordinación Nacional de Investigación, La Paz, Bolivia.

**Keywords:** HIV, Latin America, pharmacogenetics, CYP2B6, HLA-B*57:01

## Abstract

The use of combined antiretroviral therapy (cART) has resulted in a remarkable reduction in morbidity and mortality of people living with HIV worldwide. Nevertheless, interindividual variations in drug response often impose a challenge to cART effectiveness. Although personalized therapeutic regimens may help overcome incidence of adverse reactions and therapeutic failure attributed to host factors, pharmacogenetic studies are often restricted to a few populations. Latin American countries accounted for 2.1 million people living with HIV and 1.4 million undergoing cART in 2020-21. The present review describes the state of art of HIV pharmacogenetics in this region and highlights that such analyses remain to be given the required relevance. A broad analysis of pharmacogenetic markers in Latin America could not only provide a better understanding of genetic structure of these populations, but might also be crucial to develop more informative dosing algorithms, applicable to non-European populations.

## Introduction

Since HIV discovery in the early 1980`s, more than 20 antiretrovirals (ARVs) have been developed, and combined antiretroviral therapy (cART) has provided a remarkable decrease in mortality and morbidity of people living with HIV/Aids (PLHA), turning HIV infection into a chronic manageable condition (Palella *et al.*, 1998; Tseng *et al.*, 2015). 

Along with the development of new ARVs, several studies have been conducted to investigate their pharmacokinetics and possible impacts of genetic variations on treatment efficacy and safety. Indeed, polymorphisms in genes from ADME class (absorption, distribution, metabolism and excretion) have been consistently associated to cART safety and efficacy on virological control and CD4 recovery (Michaud *et al.*, 2012; Mattevi and Tagliari, 2017; Yu *et al.*, 2021). In addition to ADME class, genes related to immunological response have also been investigated in the context of CD4 recovery and hypersensitivity reactions (Mallal *et al.*, 2008; Chaponda and Pirmohamed 2011; Rajasuriar *et al.*, 2012). However, as observed in the context of many human diseases, such studies are often restricted to a few populations, and this strategy also restricts the use of any dosing algorithm that might be developed. 

According to UNAIDS data, 37.7 million people were globally living with HIV in 2020. Latin American countries accounted for 2.1 million PLHA, and about 1.4 million (65%) were accessing treatment (UNAIDS, 2021). Attempting to assess the state of art of HIV pharmacogenetics in Latin American countries, a detailed literature search on PubMed and EMBASE was conducted using non-specific keywords such as “HIV”, “polymorphism” and “country name” or “Latin America” to find as many studies as possible. Although this initial search has retrieved 1,062 articles published until February, 2022, less than 40 articles were selected for complete analysis and discussion after discarding those unrelated to the main subject, reviews and editorials. 

This review begins highlighting the association of HLA-B*57:01 allele and hypersensitivity reactions to the reverse transcriptase inhibitor abacavir (ABC), which is considered a model of successful implementation in clinical practice. In the following items, the impact of ADME variations on ARVs plasma levels and response to therapy is discussed. Polymorphisms in genes encoding cytokines and other genes indirectly related with response to cART are also described. 

## HLA-B*57:01 Screening and hypersensitivity reactions to Abacavir: A successful example of translation from basic research to clinical practice

The association between HLA-B*57:01 allele and hypersensitivity reactions (HSR) to ABC (Mallal *et al.*, 2008) is a relevant example of implementation of pharmacogenetics in clinical routine. Although ABC is generally well tolerated, 5-8% of patients experience HSR during the first 6 weeks of treatment. Clinical manifestations include mild symptoms such as fever, rash and nausea, but may also include multi-organ failure and anaphylaxis (Hewitt, 2002), leading to hospitalization and even death. The predictive effect of HLA-B*57:01 for HSR to ABC has been observed and validated using association analyses and clinical trials, followed by cost-effectiveness analyses (Lucas *et al.*, 2007; Mallal *et al.*, 2008). Screening for the HLA-B*57:01 allele prior to ABC prescription is currently recommended by the US Food and Drug Administration, in addition to health and regulatory agencies from Europe, Canada and Japan (PharmGKB). 

Studies from Latin American countries have focused on the description of allele frequencies or at least frequency of HLA-B*57:01 carriers. Since sequencing-based HLA genotyping is still expensive and laborious, different methods have been developed to detect HLA-B*57:01 presence, regardless the genotype. In such cases, the authors can only define frequency of HLA-B*57:01carriers, but not the exact allele frequency. In Brazil, a retrospective study of 96 HIV positive individuals treated with ABC and 234 HIV negative individuals from Pernambuco State, at Northeast region, has found allele frequencies of 1.5 and 1.7% respectively. The three patients carrying HLA-B*57:01 allele presented symptoms of HSR to ABC (Crovella *et al.*, 2011). Later, a study of 517 individuals from Central West region has showed a frequency of 5.6% for HLA-B*57:01 carriers among HIV positive individuals. Although exact allele frequencies could not be determined in this study, HSR to ABC was investigated, and results showed a significantly higher incidence among HLA-B*57:01 carriers (Araújo *et al.*, 2014). According to the Allele Frequency Net Database, frequencies of this allele vary across Brazilian regions, ranging from 0.005 - 0.026 in Brazilian Southeast and 0.03 among Puyanawas, from the North region (Gonzalez-Galarza *et al.*, 2020).

**Figure 1.**
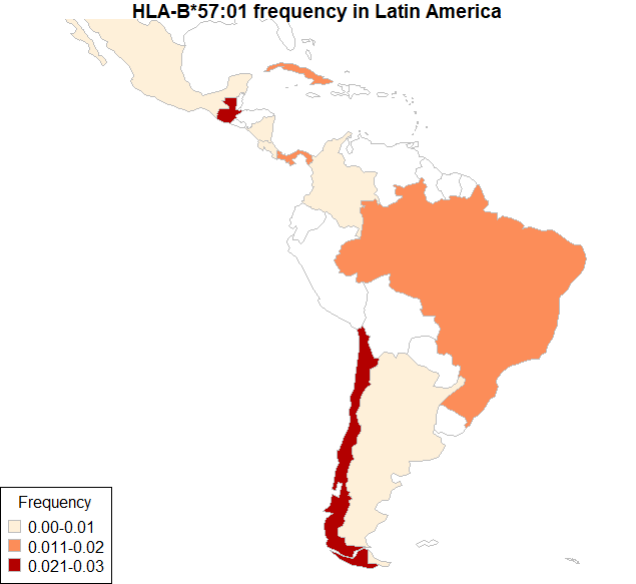
Frequency of HLA-B*57:01 allele in Latin American countries. Frequencies were retrieved from the Allele Frequency Net Database (http://www.allelefrequencies.net/). Data were available for Argentina, Brazil, Chile, Colombia, Costa Rica, Cuba, Guatemala, Mexico, Nicaragua and Panama. When more than one frequency was provided for a single country, mean values were determined.

Among Chileans, HLA-B*57:01 allele frequencies of 1.1 and 1.8% were observed respectively for HIV positive individuals and for the general population (Poggi *et al.*, 2010). Similar data were obtained for Mexican mestizos (2% and 1% for HLA-B*57:01 carriers and allelic frequency, respectively) (Sanchez-Giron *et al.*, 2011). Higher frequencies of HLA-B*57:01 carriers (5 and 4.9%,) were detected among 200 healthy individuals from Costa Rica and 1,646 HIV positive Argentinians, respectively (Arrieta-Bolaños *et al.*, 2014; Moragas *et al.*, 2015), while a prevalence of 2.7% was found in Colombian HIV-infected individuals (Martínez Buitrago *et al.*, 2019). Figure 1 summarizes the frequency of HLA-B*57:01 allele for all Latin American countries available at the Allele Frequency Net Database (Gonzalez-Galarza *et al.*, 2020).

Although all studies acknowledge that HLA-B*57:01 frequency varies according to ethnicity, remaining higher among those with European ancestry, most of them highlight the importance of a screening for this allele before prescribing ABC. Despite the lack of well-structured and statistically powered association studies performed in each country, the results available from other populations have provided strong evidence of a clear genotype-phenotype correlation (Mallal *et al.*, 2008; Ostrov *et al.*, 2012; Norcross *et al.*, 2012), suggesting that the predictive value of HLA-B*57:01 would be less prone to variations according to genetic structure of each population. 

According to technical notes and information obtained directly from local authorities, the HLA-B*57:01 screening is mandatory for patients eligible for ABC use in several Latin American countries including Argentina, Brazil, Chile, Costa Rica, Ecuador, Mexico, and the test has also been implemented in public health systems from a few countries (Ministerio de Salud - Chile, 2013; Moragas *et al.*, 2014; Instituto Mexicano del Seguro Social, 2017; Ministério da Saúde - Brasil, 2018; Ministerio de Salud Pública del Ecuador, 2019). 

## Metabolism enzymes and related transcription factors

**Figure 2.**
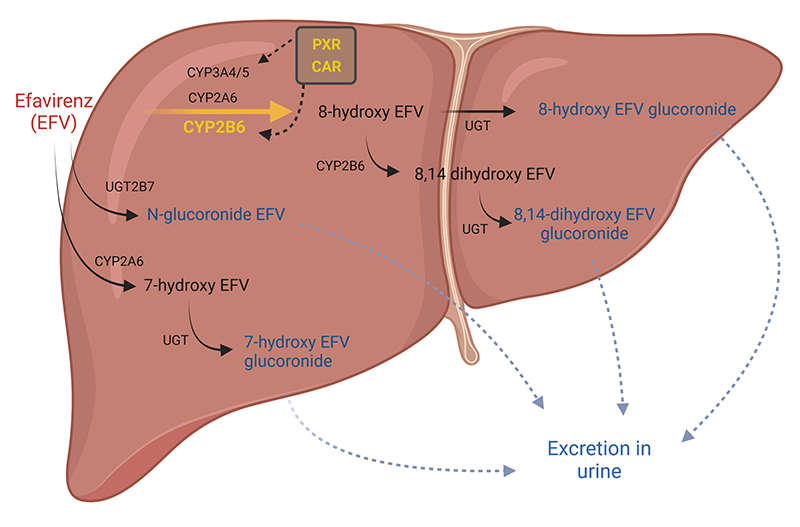
Efavirenz hepatic metabolism and pharmacogenetic associations in Latin American populations. CYP2B6 acts on primary efavirenz (EFV) metabolism pathway, converting EFV to 8-hydroxy EFV. Variations at *CYP2B6* gene have been extensively associated with EFV plasma levels and also to adverse reactions to this antiretroviral. Other enzymes (CYP3A4, CYP3A5, CYP2A6) can also generate 8-hydroxy-EFV. Transcription factors PXR and CAR regulate expression of CYP2B6 and CYP3A enzymes, and may indirectly influence EFV metabolism. Polymorphisms at genes encoding PXR and CAR were associated to EFV exposure and virological response. Alternative pathways include hydroxilation by CYP2A6 and direct glucoronidation by UGT2B7. Intermediate metabolites are then glucoronidated before excretion in urine. Genes associated with EFV pharmacokinetics and/or response to EFV in Latin American populations are shown in yellow. Created with BioRender.com.

Among metabolism enzymes, the impact of CYP2B6 variations on efavirenz (EFV) pharmacokinetics and/or response to therapy has been widely investigated. EFV is a non-nucleoside reverse transcriptase inhibitor which frequently causes central nervous system (CNS) adverse effects such as dizziness, nightmares, anxiety and depression (Kenedi and Goforth, 2011). Since EFV is metabolized mostly by CYP2B6 (Figure 2), single nucleotide polymorphisms (SNPs) at *CYP2B6* gene have been consistently associated to EFV exposure, and slow metabolizers have increased risk of adverse reactions. Although a major effect is suggested for *CYP2B6* +516G>T (rs3745274), composite genotypes including two other variations are apparently better predictors of CYP2B6 metabolic profile. Notably, data from ethnically diverse populations have showed that this association varies according to genetic ancestry (Haas *et al.*, 2004; Holzinger *et al.*, 2012), reinforcing the idea that validation of the effect in each relevant population is still required before clinical implementation.

A study with a main cohort from Haiti, and a replication sample of African Americans, has confirmed the association between *CYP2B6* +516G>T and increased EFV levels among individuals from African descent (Leger *et al.*, 2009). A study of *CYP2B6* SNPs in Chileans showed higher EFV levels among +516TT carriers, and composite genotypes including rs10403955, rs2279345 and rs8192719 as tag SNPs were even more informative for EFV levels above the minimum toxic concentration (Carr *et al.*, 2010). Similar results were obtained in another cohort from the same country, where +516G>T polymorphism was also associated to EFV levels (Cortes *et al.*, 2013). 

In Brazil, a study with a cohort from the Amazon region has found an association between +516TT genotype and lower CD4 T cell counts, while no association was observed for viral loads (Queiroz *et al.*, 2017). Notably, the impact of this variation on HIV viral loads was investigated in the whole cohort, which also included individuals using protease inhibitors instead of efavirenz. Results of two retrospective cohort studies have also showed no association between *CYP2B6* +516G>T and virological response in cohorts from the Northeast (Coelho *et al.*, 2013) and Southeast (De Almeida Velozo *et al.*, 2021) regions. The latter has also investigated the effect of composite genotypes and no association was observed either before or after adjustment for covariates, including genetic ancestry. Similarly, functional *CYP2B6* variants were not associated with virological response in a cohort from Haiti after analyses considering single SNPs, composite genotypes or CYP2B6 metabolic profile (Haas *et al.*, 2014).

Regarding adverse reactions to EFV, results obtained to date are controversial in some aspects, suggesting an impact of population substructure or heterogeneity across the different study designs. A study from the Brazilian southeastern region has found no association between *CYP2B6* polymorphisms and intolerance to EFV-containing regimens (Arruda *et al.*, 2016). Similarly, +516G>T was not associated to self-reported CNS adverse effects to EFV in a small cohort (N = 50) from the Brazilian southern region (Müller *et al.*, 2017). Despite the higher sample size, limitations of the study from the Southeast region included analysis by drug class, implying that EFV and nevirapine-containing regimens could not be dissociated, and the use of a non-specific outcome “intolerance” (Arruda *et al.*, 2016). To overcome such limitations, an independent cohort from Rio de Janeiro was recruited and only patients undergoing EFV-containing regimens were investigated. In this study, the authors have found an association between CYP2B6 slow metabolizers and increased risk of adverse effects to EFV, including either all effects or CNS adverse effects (de Almeida*et al.*, 2018). Data analyses were also adjusted for genetic ancestry to control for confounding. Recently, +516G>T was significantly associated to CNS adverse effects to EFV in a small (N=38) Chilean cohort (Poblete *et al.*, 2021). Prevalence of +516T was also determined for a cohort from Argentina (38.2%), although no association analysis was performed (Galván *et al.*, 2012).

Four studies have also performed broader characterizations of EFV pharmacogenetics, including not only *CYP2B6*, but also candidate genes related to secondary metabolism pathways (*CYP2A6*, *CYP3A4*, *CYP3A5*) and the transcription factors PXR and CAR, encoded by *NR1I2* and *NR1I3* genes. Among Haitians, variations at CYP2A6 and CYP3A4/5 enzymes were not associated to EFV exposure or virological response (Leger *et al.*, 2009; Haas *et al.*, 2014). In Brazil, these 5 additional genes were not associated with adverse reactions to EFV (de Almeida*et al.*, 2018). Nevertheless, variations in *NR1I2* and *NR1I3* were clearly associated to virological response, and the most prominent effect was observed for SNP rs2307424 (*NR1I3*), which was associated with increased virologic response after 12 months of cART. Haplotypes carrying allele rs2307424A were associated to this outcome as well (De Almeida Velozo *et al.*, 2021). Notably, SNP rs2307424 has been previously associated to EFV exposure among Chileans (Cortes *et al.*, 2013). Figure 2 summarizes the pathways for EFV primary metabolism in addition to the main genetic associations observed in Latin American countries.

In addition to EFV, genes encoding metabolism enzymes have also been investigated in Latin American populations in the context of responses to protease inhibitors (PIs). A study of 98 Brazilian HIV+ men undergoing cART regimens showed that CYP3A5*3 and CYP3A5*6 alleles do not affect plasma concentrations of lopinavir and ritonavir (Estrela *et al.*, 2008). Moreover, polymorphisms at *NR1I2* were not associated to lopinavir levels in a cohort of 38 perinatally infected children from Argentina (Bellusci *et al.*, 2013). Studies of adverse reactions to PIs have showed that UGT1A1*28 allele was associated with increased risk of atazanavir related hyperbilirubinemia among Brazilians (Turatti *et al.*, 2012) and Chileans (Poblete *et al.*, 2021). The study by Arruda *et al.* (2016) has also investigated the role of metabolism enzymes in intolerance to PIs, but no association was found. The main findings regarding association between ADME genes and response to antiretrovirals in Latin American countries were summarized in Table 1.

**Table 1.** Associations between ADME genes and response to antiretroviral therapy in Latin American populations.

Gene	Outcome	Population	Reference
Metabolism enzymes			
*CYP2B6*	Higher efavirenz levels	Haiti	Leger *et al.*, 2009
		Chile	Carr *et al.*, 2010; Cortes *et al.*, 2013
	Lower CD4 T cell counts	Brazil	Queiroz *et al.*, 2017
	Adverse effects to efavirenz	Brazil	de Almeida*et al.*, 2018
	CNS adverse effects to efavirenz	Chile	Poblete *et al.*, 2021
*NRI12* and *NR1I3*	Virological response	Brazil	de Almeida Velozo *et al.*, 2021
*NR1I3*	Efavirenz exposure	Chile	Cortes *et al.*, 2013
*UGT1A1*	Atazanavir related hyperbilirubinemia	Brazil	Turatti *et al.*, 2012
		Chile	Poblete *et al.*, 2021
Drug transporters			
*ABCB1*	Lopinavir plasma levels	Argentina	Bellusci *et al.*, 2013
	Virological failure of PI containing regimens	Brazil	Coelho *et al.*, 2013
	Decreased immunological response to efavirenz	Brazil	Coelho *et al.*, 2018
*ABCC1*	Virological failure of PI containing regimens	Brazil	Coelho *et al.*, 2013
*ABCC2*	Intolerance to PI containing regimens	Brazil	Arruda *et al.*, 2016
*SLCO1B1*	Trough plasma concentration of lopinavir	Brazil	Kohlrausch *et al.*, 2010
	Intolerance to NRTIs	Brazil	Arruda *et al.*, 2016

ADME: absorption, distribution, metabolism and excretion. PI: protease inhibitors. NRTIs: nucleoside reverse transcriptase inhibitors.

### Drug transporters

Genes encoding drug transporters are also important targets for HIV pharmacogenetics due to their role in ARVs influx and efflux from different cells, regulating bioavailability and penetration in target cells and viral sanctuary sites. 

Among ABC transporters (ATP-binding cassette proteins), the *ABCB1* gene - which encodes P-glycoprotein - has been widely investigated in response to different ARVs, including EFV and PIs (Michaud *et al.*, 2012). Variations at this gene were not associated with plasma concentrations or virological response to EFV among Haitians (Leger *et al.*, 2009; Haas *et al.*, 2014). In Brazil, *ABCB1* variations were not associated with intolerance or CNS adverse reactions to EFV (Arruda *et al.*, 2016; de Almeida*et al.*, 2018) in cohorts from Southeast region, while 1236C>T was associated with decreased immunological response to this antiretroviral in patients from Northeast region (Coelho *et al.*, 2018).

Conflicting results were also observed in response to protease inhibitors, suggesting that *ABCB1* effect may be influenced by population substructure. *ABCB1* genotypes for 1236C>T, 2667G>T/A and 3435C>T were not predictors of lopinavir and ritonavir concentrations in plasma, semen or saliva of 113 HIV positive individuals (Estrela *et al.*, 2009). By contrast, SNP 3435C>T was associated with virological failure of PI containing regimens (Coelho *et al.*, 2013). In Argentinian cohorts, *ABCB1* polymorphisms were associated to lopinavir plasma levels in HIV-1 perinatally infected children (Bellusci *et al.*, 2013). Association to porphyria cutanea tarda was also suggested, although it was not clear whether this condition was mostly influenced by HIV infection or treatment (Pagnotta *et al.*, 2020). Other ABC transporters have also been investigated in response to cART. In Brazil, *ABCC1* and *ABCC2* genes were respectively associated to virological failure (Coelho *et al.*, 2013) and intolerance to PI containing regimens (Arruda *et al.*, 2016). 

Among solute carrier transporters, a *SLCO1B1* polymorphism was associated with the trough plasma concentration of lopinavir, considering a cohort composed of 99 individuals treated with lopinavir and ritonavir for at least 4 weeks (Kohlrausch *et al.*, 2010). Later, the same gene was associated with intolerance to nucleoside reverse transcriptase inhibitors (Arruda *et al.*, 2016). Both studies were conducted among HIV positive individuals from Brazilian Southeast. 

### Other associations

In addition to ADME genes, association studies have also investigated a role for genes related to specific phenotypes, such as metabolic outcomes. In Brazil, polymorphisms in *SCAP*, *APOE*, *APOA5* and *ADIPOR2* genes were associated with high levels of triglycerides and cholesterol in individuals using protease inhibitors (Lazzaretti *et al.*, 2013; Castilhos *et al.*, 2015), while a polymorphism in *APM1* influenced adiponectin levels (Trinca *et al.*, 2010). *ESR1* and *ESR2* genes were both associated with body mass index (BMI) and total subcutaneous fat, while *ESR2* also conferred risk for lipoatrophy in women undergoing PI-containing regimens (Gasparotto *et al.*, 2012). SNPs at *APOA5*, *APOC3* and *SIK3* were also implicated in risk of hypertriglyceridemia in a case-control study including 602 Mexicans patients receiving PIs (Bautista-Martínez *et al.*, 2022). 

Additional studies have also investigated the role of polymorphisms in genes encoding cytokines and restriction factors and response to cART. In Brazil, variations at CCR5Δ32 (Rigato *et al.*, 2008), *IL2* (Coelho *et al.*, 2018) and *IL18* (Andrade-Santos *et al.*, 2019) were associated to CD4+ T-cell recovery. Moreover, the *IL10* −1082 AA genotype was associated with allergic reactions to efavirenz (Rodrigues *et al.*, 2014). A large study, including 873 participants from United States and Puerto Rico, have also showed an association between polymorphisms in genes encoding TNF-α, TRAIL, Bcl-2, IL-15, IL-15Rα and IFN-α were associated CD4 lymphocyte counts after long-term cART. However, data analyses were not stratified by country, and data specific from Puerto Rico subjects were not available (Haas *et al.*, 2006).

## Conclusions

Pharmacogenetic analysis is a promising path to enable implementation of personalized regimens that might prevent cART unfavorable outcomes such as adverse reactions to ARVs. However, universal application of genetic tests may be challenging not only due to budget limitations of each country, but also due to the lack of validation in ethnically diverse populations. Indeed, large scale pharmacogenetics studies including participants with varying genetic backgrounds are still scarce, and populations such as Latin Americans are underrepresented. 

In this review, we summarize the main data regarding HIV pharmacogenetics obtained from studies focused on Latin American cohorts. The literature search clearly showed that most studies were conducted among Brazilians, with additional analyses from Haiti, Chile, Argentina, Costa Rica, Mexico and Puerto Rico. The scarcity of studies focusing on this region raises concern. Despite the geographical proximity, allele frequencies may be remarkably different within Latin America populations since most of them may exhibit complex genetic backgrounds. Unlike the example of HLA-B*57:01 screening to prevent HSR to ABC - which is probably informative for any population - genetic associations described for Europeans or clearly defined ancestry groups are rarely generalizable to genetically complex populations. Further analyses of Latin American cohorts along with other understudied populations are crucial to improve fine mapping of causative variations and to ultimately develop more effective dosing algorithms, applicable to diverse ethnicities. 
